# Trajectory and Determinants of Left Ventricular Dysfunction Following Anthracycline-Based Therapy Among Breast Cancer Patients: A Three-Year Cohort Analysis in Indonesia

**DOI:** 10.3390/ph19060901

**Published:** 2026-06-06

**Authors:** Nadea Olyvia Wardani, Risani Andalasia Putri, Lia Amalia, Nadia Hanum, Erwin Martinez Faller, Ernieda Hatah, Zulfan Zazuli

**Affiliations:** 1 Doctoral Program in Pharmacy, School of Pharmacy, Institut Teknologi Bandung, Bandung 40132, Indonesia; nadeaolyviaward@gmail.com; 2 Department of Pharmacy, Dharmais National Cancer Center Hospital, Jakarta 11420, Indonesia; risani.andalasia@dharmais.co.id; 3 Department of Pharmacology–Clinical Pharmacy, Institut Teknologi Bandung, Bandung 40132, Indonesia; lia_amalia@itb.ac.id (L.A.); nadia.hanum@itb.ac.id (N.H.); 4 College of Allied Health Sciences, Holy Child Central Colleges Inc., Surallah 9512, Philippines; erwinfaller@hccci.edu.ph; 5 Center for Quality Management of Medicines, Faculty of Pharmacy, Universiti Kebangsaan Malaysia, Kuala Lumpur 50300, Malaysia; ernieda@ukm.edu.my; 6 Center of Excellence for Innovative Cosmeceuticals and Natural Medicines for Degenerative Disease, Center for Pharma Valorisation, School of Pharmacy, Institut Teknologi Bandung, Bandung 40132, Indonesia

**Keywords:** cardiotoxicity, LVEF, doxorubicin, trastuzumab, breast cancer

## Abstract

**Background**: Doxorubicin and trastuzumab are effective breast cancer therapies but carry cardiotoxicity risk, indicated by decreased left ventricular ejection fraction (LVEF). This study aimed to analyze LVEF decline trends, cardiotoxicity incidence, and associated risk factors in breast cancer patients receiving doxorubicin with or without trastuzumab at Dharmais National Cancer Center Hospital, Jakarta. **Methods**: A retrospective cohort study was conducted using medical records of female patients aged ≥18 years with serial echocardiography (baseline and ≥1 follow-up) and ≥3 years of follow-up. Cardiotoxicity was defined as an absolute LVEF decline of ≥10% from baseline to <53%. We used the Mann–Whitney U test for LVEF trends, the Chi-square or Fisher’s exact for risk factor associations, and Firth’s Cox proportional hazards model for relative risk estimation. **Results**: Among the 301 patients, LVEF declined significantly at the fourth and sixth chemotherapy cycles and remained reduced during follow-up beyond 3 years. Cardiotoxicity occurred in 20 patients (6.65%), while 72 patients (23.92%) had borderline low LVEF. Significant risk factors for cardiotoxicity were continued trastuzumab-based chemotherapy (_adj_HR 3.79; 95% CI 1.39–9.23) and a history of hypertension (_adj_HR 0.2; 95% CI 0.02–0.81). This apparent protective effect of hypertension is likely due to the concurrent use of cardioprotective anti-hypertensive medications in this subgroup. **Conclusions**: Intensive cardiac monitoring is essential for breast cancer patients receiving sequential trastuzumab therapy, especially those with cardiovascular risk factors. Additional study with extended monitoring, more sensitive cardiac parameters, and detailed evaluation of concurrent cardioprotective therapies are warranted.

## 1. Introduction

Globally, breast cancer is the second most frequently diagnosed cancer and the most common cancer type among women [1], accounting for approximately 12% of all cases worldwide. In 2022, the World Health Organization’s GLOBOCAN data reported approximately 2.3 million new breast cancer cases and 670,000 deaths globally. In Indonesia, this burden is particularly prominent, with 209,748 new cases recorded over the last five years, representing 20.6% of all cancer cases in the country [1]. Nonetheless, the impact of breast cancer varies significantly across regions [2]. Evaluating breast cancer mortality rates in conjunction with incidence rates offers a valuable insight into the severity of breast cancer diagnoses for women across different nations [3]. GLOBOCAN data covering 185 countries demonstrate marked disparities by development level, with 17 deaths per 100 breast cancer diagnoses in very high-HDI countries, compared with 56 deaths per 100 diagnoses in low-HDI countries [4]. The apparent difference in the study’s global patterns and trends in breast cancer incidence and mortality across 185 countries is possibly linked to inequalities in early detection, prompt diagnosis, and access to thorough breast cancer care.

Doxorubicin is an anthracycline antibiotic commonly employed in chemotherapy for addressing solid tumors and hematogenous malignancies, particularly in the treatment of breast cancer [5]. For patients whose tumors overexpress human epidermal growth factor receptor 2 (HER2/ErbB2), the incorporation of trastuzumab into treatment regimens has led to substantial improvements in therapeutic response and survival outcomes [6]. In the adjuvant setting, trastuzumab has been shown to significantly increase objective response rates and extend overall survival when compared with chemotherapy alone, translating into meaningful clinical benefit for patients [7]. However, from a patient-centered perspective, these therapeutic gains are counterbalanced by a clinically relevant risk of cardiotoxicity, particularly when trastuzumab is administered in combination with or following anthracycline exposure [8]. Cardiac dysfunction associated with these treatments not only affects clinical outcomes but also has a measurable negative impact on patient-reported outcomes, including physical functioning, fatigue, and overall health-related quality of life, potentially leading to treatment interruption or discontinuation [9].

Cardiotoxicity encompasses direct structural myocardial damage and indirect effects via thrombogenic states or hemodynamic shifts [10]. Risk factors include age, obesity, hypertension, diabetes, and pre-existing cardiac dysfunction [11]. Mechanistically, anthracyclines and trastuzumab drive cardiotoxicity by inducing cellular apoptosis through topoisomerase II beta (TOP2β) inhibition and ROS-mediated mitochondrial dysfunction. Specifically, doxorubicin facilitates iron mobilization, creating a self-amplifying cycle of free radical generation. This severe oxidative stress structurally degrades essential organelles—including the mitochondria, nucleus, and endoplasmic reticulum—which depletes intracellular calcium and ultimately impairs cardiac contractility [12,13,14,15,16]. Diagnostically, while early criteria defined cancer therapeutics-related cardiac dysfunction (CTRCD) at an LVEF threshold of 55% [17], current European Society of Cardiology (ESC) guidelines define it as an absolute LVEF reduction of >10 percentage points from baseline to a value of <53% [18].

The significance of evaluating the administration of doxorubicin and trastuzumab chemotherapy is underscored by the growing number of breast cancer survivors and adverse cardiovascular events [19]. The Asian cohort examined in Taiwan exhibited a cumulative incidence of significant cardiovascular events of approximately 3.7% [20]. The incidence of trastuzumab-related cardiac dysfunction in Asian breast cancer patients was reported to be around 7.4% in Korean research [21]. Consequently, early detection and assessment of cardiotoxicity risk are crucial for enabling physicians to modify the chemotherapy regimen, thereby maximizing therapeutic effectiveness while minimizing the potential for side effects [8]. However, there are no data representing the Southeast Asian population. Therefore, this study is necessary to classify cardiotoxicity based on the latest guidelines. This study aimed to analyze the trend of LVEF decline, the incidence of cardiotoxicity, and the associated risk factors in breast cancer patients receiving doxorubicin with or without trastuzumab at Dharmais National Cancer Center Hospital, Jakarta.

## 2. Results

**Figure 1:**
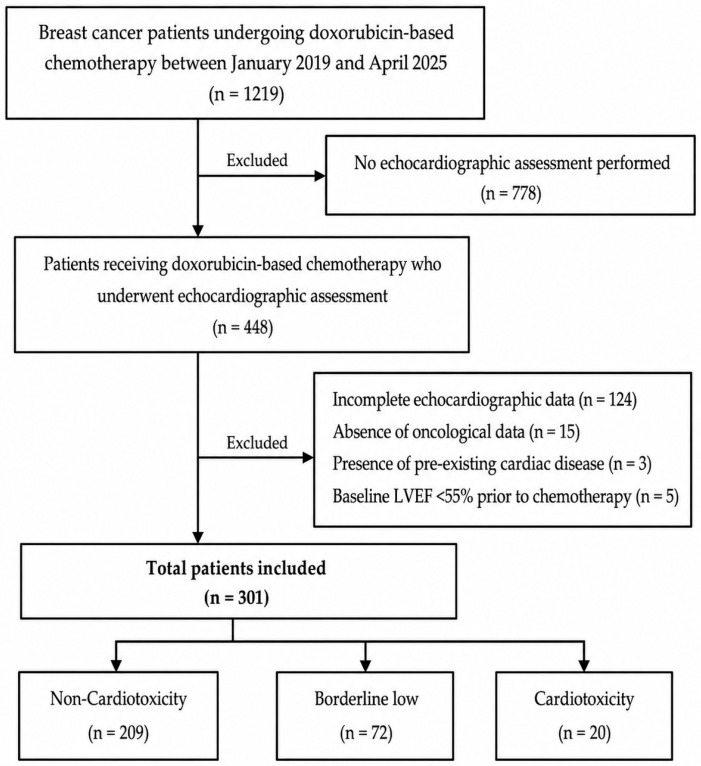
Flowchart of Study Subject Selection.

From January 2019 to April 2025, a total of 1219 breast cancer patients underwent chemotherapy with the doxorubicin regimen. Of these patients, 448 attended the cardiology clinic and received LVEF assessment using echocardiography, both before and throughout treatment. A total of 147 patients were excluded, with the following details: 124 patients lacked LVEF values prior to chemotherapy (baseline), and incomplete medical records. Additionally, 15 patients were missing histologically confirmed breast cancer data, 3 patients had pre-existing heart disease, and 5 patients exhibited LVEF values below 55% before chemotherapy. Consequently, a total of 301 patients fulfilled the inclusion and exclusion criteria. Figure 1 displays the flowchart depicting the selection and screening process utilized in this investigation.

All patients included in the study received Doxorubicin-based chemotherapy, The summary of patients’ characteristics is shown in Table 1. The median age was 47 years (IQR 41.25–53), with majority (n = 221, 73.42%) aged between 40–60 years. The majority of patients exhibited a body mass index (BMI) within the normal range (n = 138, 45.85%), followed by those classified as overweight (n = 114, 37.87%) and obese (n = 38, 12.62%). The median (IQR) of body surface area (BSA) was 1.54 m^2^ (1.47–1.64). The predominant ethnic groups were Javanese (n = 87, 28.90%), Sundanese (n = 85, 28.24%), and Betawi (n = 79, 26.25%). A very small percentage of patients reported a history of smoking (n = 1, 0.33%).

The majority of patients in the study were diagnosed with stage III breast cancer (n = 147, 48.84%), followed by stage II (n = 106, 35.22%) and stage IV (n = 38, 12.62%). Left-sided tumors occurred with greater frequency, accounting for n = 153, 50.83% of cases. Hormone receptor positivity was noted in n = 147, 47.51% for estrogen receptors (ERs) and n = 134, 44.52% for progesterone receptors (PRs), with HER2 positivity identified in n = 123, 40.86% of cases. Triple-negative breast cancer (TNBC) represented n = 69, 22.92% of cases. Almost all patients were administered granulocyte colony-stimulating factor (n = 299, 99.34%) and underwent radiation therapy (n = 211, 70.10%). The predominant chemotherapy regimen utilized was Fluorouracil, Adriamycin/Doxorubicin, Cyclophosphamide (FAC), accounting for n = 251 (83.39%) of cases, and regimen with AC was noted in n = 50, 16.61%. A cumulative median doxorubicin dose of range between 300–360 mg/m^2^ (n = 258, 85.71%). A minority of patients received subsequent chemotherapy regimens after receiving FAC/AC, specifically Taxane, Cyclophosphamide, Trastuzumab (TCH) n = 30, 9.97% or Taxane + Trastuzumab (TH) n = 4, 1.33%.

The baseline median LVEF was 65.6% (IQR 62.3–69). Hypertension was the most prevalent clinical comorbidity at n = 82, 27.24%, followed by dyslipidaemia at n = 11, 3.65%, and diabetes mellitus at n = 7, 2.33%. This cohort did not exhibit cardiac comorbidities such as atrial fibrillation or chronic kidney disease. A limited percentage of patients indicated a history of percutaneous coronary intervention (n = 2, 0.66%). In terms of concomitant therapies, n = 21, 6.98% of patients received β-blockers, n = 45, 14.95% were administered angiotensin converting enzyme inhibitors (ACEIs) or angiotensin receptor blockers (ARBs), n = 11, 3.65% were prescribed statins, and a total of n = 109, 36.21% received cardioprotective agents during chemotherapy, such as ACEI/ARB, β-blockers.

**Figure 2:**
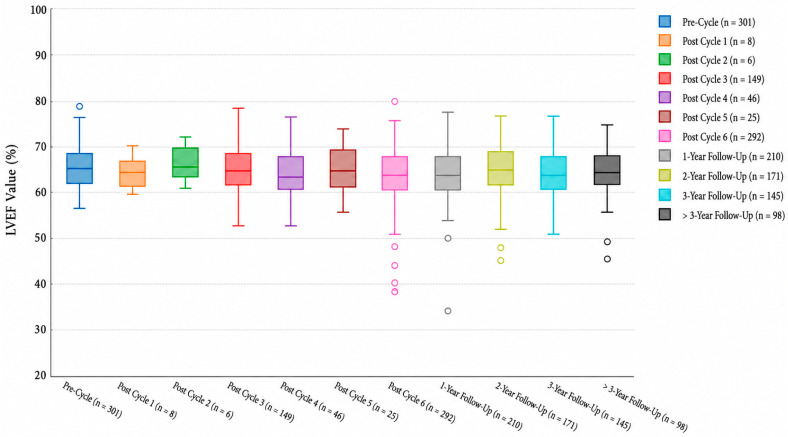
Longitudinal comparison of LVEF across chemotherapy cycles. Note: the *p*-value of each cycle were generated with comparison to the LVEF pre-cycle or baseline value as reference.

The median LVEF was approximately 65% (IQR 62–69%) at baseline (pre-cycle), suggesting that systolic function was preserved prior to the initiation of chemotherapy. A modest reduction in LVEF of 2.6% (IQR) was noted after consecutive cycles of doxorubicin-based treatment. By cycle 2, the median LVEF exhibited a modest decline, a tendency that persisted through cycles 4 and 6. The variability in LVEF values increased, as indicated by the extended IQR and the emergence of outliers below 50%, which signify early cardiotoxic consequences in a subset of individuals. Statistical analysis revealed substantial differences from baseline at multiple intervals, notably after cycle 4, cycle 6, and at the 1-year follow-up (*p* < 0.05). These results suggest that progressive deterioration in cardiac function is associated with cumulative anthracycline exposure. A comparison of LVEF values before and after each chemotherapy cycle is shown in Figure 2.

Patients’ characteristics among three groups: non-cardiotoxicity (n = 209; 69.43%), borderline low LVEF (n = 72; 23.92%), and cardiotoxicity (n = 20; 6.65%) were compared and presented in Table 2. No significant differences were observed across demographic factors and tumor profiles. Several clinical and treatment-related factors were associated with outcomes of cardiotoxicity. Subsequent chemotherapy, especially TCH regimens, was associated with an increased incidence of cardiotoxicity (*p* = 0.045). Trastuzumab exposure showed a trend toward increased cardiotoxicity risk; however, the association did not reach statistical significance. Patients who completed ≥18 cycles or partial trastuzumab cycles tended to have a higher incidence of cardiotoxicity compared with those who did not receive trastuzumab therapy (*p* = 0.057). Baseline LVEF exhibited significant differences among groups (*p* < 0.001), with patients experiencing cardiotoxicity presenting lower baseline values. From a comorbidity perspective, patients with hypertension (*p* = 0.029) and dyslipidaemia (*p* = 0.003) but not diabetes mellitus demonstrated statistically significant differences between groups. Analysis of medication history reveals that β-blockers, ACEI/ARB, statins, and cardioprotective therapy were not associated with cardiotoxicity event.

**Figure 3:**
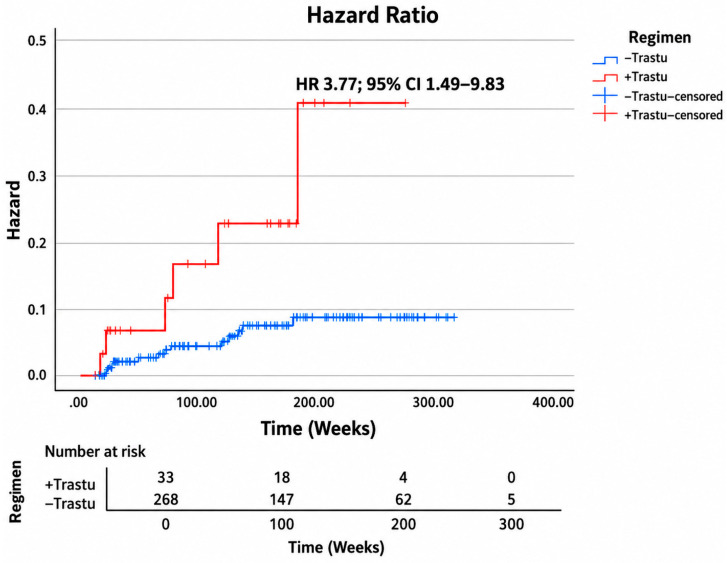
Comparison of Hazard Ratios Between Doxorubicin-based Regimens with or Without Trastuzumab.

Figure 3 shows that the cumulative hazard curve of patients who received a combination of doxorubicin followed by trastuzumab (+Trastu) was higher than that of the group without trastuzumab (−Trastu), as indicated by the results of multivariate analysis using the Cox proportional hazards model. The risk of cardiotoxicity began to increase with the commencement of therapy and persisted. The curve slope of the +Trastu group was particularly precipitous, especially up to week 150. In contrast, the Trastu group exhibited a more gradual increase in hazard, which appeared to plateau after 200 weeks. The Cox proportional hazards model quantitatively estimates the hazard ratio (HR) to compare the relative risk between two regimens. The variation in curve slope indicates that +Trastu is a significant predictor of cardiotoxicity events when controlling for other clinical covariates. The cumulative hazard in the +Trastu group exhibited a more rapid increase from the initiation of therapy until 200 weeks of observation in comparison to the −Trastu group. The steeper curve in the +Trastu group indicates a more rapid and substantial accumulation of events, even with the censoring of certain patients. The censoring distribution exhibited relative uniformity, thereby not introducing substantial bias in the hazard estimation. These findings were further supported by the Kaplan–Meier cardiotoxicity-free survival analyses, which demonstrated lower event-free survival probability among patients receiving sequential trastuzumab therapy (Supplementary Figure S1), while the overall study cohort maintained relatively high cardiotoxicity-free survival throughout longitudinal follow-up (Supplementary Figure S2). The findings indicate that the +Trastu regimen presents a relative risk 3.77 times greater than the −Trastu regimen (HR 3.77; 95% CI 1.49–9.83).

The incidence of left ventricular dysfunction (LVD) in breast cancer patients treated with doxorubicin with or without trastuzumab was consistently influenced by subsequent chemotherapy with trastuzumab, as demonstrated by multivariate analysis (Table 3). The history of subsequent chemotherapy using the TCH/TH regimen showed a significant association (_adj_HR of 3.79, a 95% CI of 1.39–9.23, and *p* = 0.01). Consequently, individuals undergoing trastuzumab treatment, regardless of the duration of cycles, had almost a twofold heightened chance of experiencing cardiotoxicity. A previous medical history of hypertension showed a significant association with an _adj_HR of 0.2; 95% CI 0.02–0.81; *p* = 0.02. This apparent protective effect in the multivariate analysis diverged from typical physiological expectations. Further analysis revealed that this divergence was largely driven by a significantly higher proportion of hypertensive patients receiving concurrent cardioprotective medications compared to normotensive patients (51.9% vs. 22.7%; *p* = 2.32 × 10^−6^), as detailed in Table 4.

## 3. Discussion

A notable reduction in LVEF was detected starting at the 4th and 6th treatment cycles and during subsequent annual evaluations. This discovery demonstrates a relationship between the total dosage of doxorubicin and its escalating harmful effects on the myocardium resulting from repeated exposure [22,23]. Investigations indicate that the average cumulative dose in the usual treatment group was around 364 mg/m^2^, with a significant increase in cardiac toxicity when the cumulative dose approached or exceeded 400–450 mg/m^2^ [24]. At this dosage, the risk of developing congestive heart failure markedly increases, highlighting the necessity for vigilant and ongoing monitoring of cardiac function during the treatment period.

The molecular mechanisms underlying doxorubicin-induced cardiotoxicity can be classified into three primary domains: oxidative stress, alterations in cell death pathways, and epigenetic alterations [16,18,25]. The mechanism underlying TIC is not yet fully elucidated. Conceptually, both medications target three pathological mechanisms: mitochondrial dysfunction, apoptosis/multiple programmed cell death, and progressive cardiac remodeling [26,27,28,29]. Doxorubicin chemotherapy, subsequent to trastuzumab, even showed higher cytosolic and mitochondrial ROS, mitochondrial membrane depolarisation, Ca^2+^ dysregulation, and more severe apoptosis in cell models [30,31,32,33].

This study found that out of 301 patients analyzed, 6.65% experienced cardiotoxicity, while 23.92% were classified in the borderline low LVEF category. This distribution is consistent with the findings of Cho et al. (2020), which indicated a cumulative incidence of cardiotoxicity of 6.1% at two years post-doxorubicin initiation without trastuzumab, rising to 20.2% with the addition of trastuzumab [34]. A study in Japan spanning 31 years demonstrated a long-term trend in anthracycline-related cardiomyopathy, with a peak incidence of 6% in the 1990s, followed by stabilization at approximately 2% in the 2010s [35]. Research conducted by Gerodias et al. (2022) across ten research centers involving 341 patients revealed that 33 individuals developed anthracycline-induced cardiotoxicity (AIC), resulting in an incidence rate of 9.68% [36]. Additionally, 9 patients (2.6%) experienced clinical heart failure. The findings indicate that the incidence of AIC in Asian populations is between 6% and 10%, especially in breast cancer patients.

This study confirms that subsequent chemotherapy (specifically TCH or TH regimens), trastuzumab cycle count, hypertension, and dyslipidaemia significantly increase the risk of LVD in doxorubicin-treated patients [37]. Trastuzumab exhibits a robust cardiotoxic profile, particularly with prolonged or combination use [38], and can induce toxicity independently of anthracyclines—especially in patients with preexisting cardiovascular risks like dyslipidaemia [37,38]. Because extended exposure accelerates the accumulation of subclinical myocardial injury leading to systolic dysfunction [39], meticulous regimen selection is critical for high-risk patients.

The findings are consistent with the meta-analysis conducted by de Azambuja et al. (2014), which indicated a potential 2.5-fold increase in the risk of congestive heart failure associated with extended trastuzumab treatment [40]. Hypertension is a significant factor in the cardiotoxicity associated with both anthracyclines and trastuzumab. Dyslipidaemia has been examined in relation to other cardiovascular risk factors, including hypertension and diabetes. Dyslipidaemia may exacerbate cardiotoxicity induced by anthracyclines [41].

The multivariate analysis utilizing the Cox proportional hazard model indicated that patients treated with a combination of doxorubicin and trastuzumab (+Trastu) exhibited a higher cumulative hazard curve in comparison to those not receiving trastuzumab (−Trastu). Due to the small number of cardiotoxic events, the standard Cox model may yield biased estimates and misleading predictive performance. Therefore, Firth’s penalized Cox proportional hazards model was applied as a robust alternative. This method effectively reduces small-sample bias and provides a more accurate and stable estimation of the relative risk, confirming the validity of our findings. In our multivariate evaluation, two variables consistently emerged as significant determinants linked to LVD: a history of subsequent trastuzumab-based chemotherapy and a pre-existing diagnosis of hypertension. This finding establishes a crucial foundation for exploring how sequential targeted therapies and comorbidities impact a patient’s vulnerability to cardiotoxicity.

A history of subsequent chemotherapy using the TCH/TH regimen was associated with an adjusted hazard ratio (_adj_HR) of 3.79. This suggests a roughly fourfold increase in the risk of developing LVD or cancer therapy-related cardiovascular dysfunction (CTRCD) in comparison to individuals who do not receive subsequent chemotherapy. This study assessed heart function in patients undergoing doxorubicin-based chemotherapy for at least 3 years. Patients with HER2 overexpression subsequently continued treatment with trastuzumab-based regimens after chemotherapy. The duration of trastuzumab therapy varied between patients, including both short-term (<18 cycles) and long-term (>18 cycles) treatment. Cardiac function was monitored regularly using echocardiography every 3 months during follow-up [42]. This association is biologically and clinically plausible; cumulative exposure to HER2-targeted therapies, intensifies myocardial stress—via oxidative damage, sarcomere dysfunction, and TOP2β disruption—diminishing contractile reserve and increasing CTRCD risk. Accordingly, the 2022 ESC Guidelines classify prior anthracycline or trastuzumab exposure as a major risk factor, mandating vigilant monitoring with serial echocardiography and cardiac biomarkers (troponin or BNP) [39].

The combination or sequential administration of chemotherapy regimens with trastuzumab elevates the risk of CTRCD. Initial clinical trials and cohort studies involving HER2+ patients have demonstrated increased rates of cardiac dysfunction associated with the concurrent or sequential administration of trastuzumab and anthracyclines [43]. In multiple phase III trials, the occurrence of “cardiac events” was as high as 27% when combined with anthracyclines but significantly lower when administered sequentially or without anthracyclines [44]. The observed _adj_HR of 3.79 for a history of subsequent chemotherapy supports the correlation between increased cumulative cytotoxic exposure and myocardial impairment, as well as the heightened risk of chemotherapy-related cardiac dysfunction.

A notable and seemingly paradoxical finding in our study was that a history of hypertension correlates with a significantly reduced risk, evidenced by an _adj_HR of 0.20, indicating diminished cardiotoxicity risk. Rather than indicating that hypertension itself is biologically protective, this result highlights a classic case of confounding by indication. As demonstrated in Table 4, patients with pre-existing hypertension were significantly more likely to be receiving concurrent anti-hypertensive therapies (51.9%)—such as ACEIs, ARBs, or β-blockers—compared to normotensive patients (22.7%, *p* < 0.001). In cardio-oncology practice, these medications are standard-of-care cardioprotectives known to mitigate anthracycline and trastuzumab-induced cardiotoxicity. Therefore, the “hypertension” variable in our model essentially acted as a proxy for the receipt of these protective cardiovascular treatments. The profound benefit of these concurrent medications masked the underlying cardiovascular risk posed by hypertension itself. This study addresses a significant research gap by presenting local evidence on the extent of hypertension’s effect on cardiotoxicity in Indonesian breast cancer patients, a topic that has been infrequently documented in prior research.

The 2022 ESC Guidelines categorize hypertension as a fundamental cardiovascular risk factor that influences the risk of CTRCD associated with both anthracycline (type 1, irreversible) and trastuzumab (type 2, reversible) treatments. The guidelines suggest the optimization of blood pressure prior to, throughout, and following chemotherapy [39]. Hypertension is included in risk stratification models to assess the intensity of monitoring (e.g., serial echocardiography/global longitudinal strain (GLS) and biomarker assessment) and preventive interventions. Recent systematic reviews indicate that hypertension elevates the odds and HR of CTRCD associated with both anthracycline and trastuzumab exposure. This risk remains significant after controlling for age, BMI, and other metabolic factors. In cohorts receiving anthracyclines, hypertensive comorbidity is consistently linked to an increased incidence of CTRCD, with analogous results noted for trastuzumab, particularly when given sequentially following anthracyclines [45,46,47].

This study has several strengths that support the validity and applicability of its findings. The retrospective design enabled a comprehensive evaluation of treatment patterns and clinical outcomes over a three-year follow-up period, providing a relatively long timeframe for assessing longitudinal trends and outcomes. Conducted at a national cancer referral center, the study involved a diverse and representative patient population, with consistent access to standardized breast cancer therapies and diagnostic facilities, enhancing data reliability. In addition, the inclusion of the borderline low LVEF category was clinically important because it may represent early or subclinical cardiac dysfunction preceding overt cardiotoxicity. According by the Common Terminology Criteria for Adverse Events (CTCAE) version 6.0 criteria, a ≥10% decline in LVEF from baseline may indicate moderate cardiac toxicity requiring closer monitoring and supports earlier cardiac surveillance in cardio-oncology practice in the future [48]. Therefore, the generalizability of the findings to healthcare settings with different characteristics and resources should still be interpreted cautiously.

However, several limitations should be noted. Potential selection bias may have occurred because only a subset of patients underwent echocardiographic evaluation, particularly during the COVID-19 pandemic period (2019–2021), resulting in incomplete serial LVEF data. Echocardiographic data were incomplete due to national insurance protocols allowing assessments only after every two chemotherapy cycles, limiting optimal cardiac function monitoring. The transition from physical to electronic medical records introduced potential data loss. Cardiac assessment was restricted to LVEF, without incorporating more sensitive parameters such as troponin, NT-proBNP, or GLS. Limited follow-up communication with patients hindered complete longitudinal data collection, potentially affecting the interpretation. Lastly, small events (n = 20) limit statistical power to detect smaller associations or adjust for many confounders.

## 4. Methods

### 4.1. Study Design and Ethical Approval

A retrospective cohort study was conducted at the Dharmais National Cancer Hospital, Jakarta, Indonesia. The study obtained ethical approval from the Institutional Health Research Ethics Committee of the Dharmais National Cancer Hospital (Approval number: DP.04.03/11.7/173/2024 August 2024).

### 4.2. Study Population

A universal sampling methods were used to include all patients who met between January 2019 and December 2021. Of 1219 eligible patients, 448 underwent serial echocardiographic LVEF assessment, and 301 fulfilled the final inclusion criteria. Patients were longitudinally monitored from a pre-chemotherapy baseline through April 2025. The follow-up framework consisted of cardiac evaluations during the initial six chemotherapy cycles, followed by comprehensive post-therapy assessments at 1, 2, 3, and >3 years. The study’s eligibility criteria: female breast cancer patients aged ≥18 years who received doxorubicin-based chemotherapy, with or without trastuzumab, histologically confirmed breast cancer, availability of at least two echocardiographic LVEF assessments using Simpson’s biplane method (one at baseline and at least one during or after treatment), and a longitudinal follow-up exceeding 3 years (data collection until April 2025). Patients with incomplete medical records (either physical or digital), pre-existing cardiac dysfunction, or a baseline LVEF of <55% were excluded.

### 4.3. Outcome Definitions and Data Collection

Cardiotoxicity was the primary outcome, as determined by LVEF evaluation. Despite efforts to standardize criteria, there remains considerable heterogeneity in definitions of cardiotoxicity, especially in relation to LVEF cutoffs. The European Society of Cardiology (ESC) establishes that the classification is divided into two main categories: Cardiotoxicity was defined as a decrease in LVEF of >10% to a value < 53%. Additionally, a borderline low LVEF was defined as a decline of >10% to a final LVEF in the range 53–54% [18,39,49]. This classification is also consistent with the reference values and clinical practice standards routinely used by cardiologists at our institution [50]. Serial echocardiographic evaluations employing Simpson’s biplane method were performed at baseline, throughout treatment, immediately post-therapy, and during medium-and long-term follow-up to identify both early- and late-onset cardiotoxic effects. Other variables analyzed as potential factors included age, clinical and oncological characteristics, cumulative doxorubicin dose, history of comorbidities (e.g., hypertension, diabetes), and medication history. To minimize the risk of measurement bias, all LVEF values were sourced from echocardiography reports verified by cardiologists.

### 4.4. Statistical Analysis

Statistical analyses included the Mann–Whitney U test to assess LVEF decline and the Chi-square or Fisher’s exact test, to evaluate associations between clinical factors and cardiotoxicity. Variables with statistical significance in the univariable and bivariable analyses, along with clinically relevant covariates based on prior cardio-oncology literature, were considered for multivariable analysis. Given the limited number of cardiotoxicity events, variable selection was restricted to minimize overfitting and maintain model stability. Collinearity among covariates was assessed before analysis, and no significant multicollinearity was identified. Firth’s Cox proportional hazards model was applied to reduce small-sample bias associated with rare-event survival data. Although stratification of LVEF decline events identified borderline low LVEF (<55%) as a marker of subclinical LVD, this criterion does not meet the formal definition of cardiotoxicity. Therefore, in the Cox proportional regression models, patients with borderline low LVEF (53–54%) were classified as non-events (non-cardiotoxicity), while those who developed overt cardiotoxicity were classified as events. This classification approach was based on the clinical practice standards routinely used by cardiologists at our institution, in which LVEF values ≥ 53% are still considered within the normal or low-normal range [50]. Analyses were performed using R version 4.3.1, with significance defined as *p* < 0.05. In this study, multivariate analysis was not performed on all variables with a *p*-value < 0.05.

## 5. Conclusions

Significant declines in LVEF were observed during treatment and persisted into mid- and long-term follow-up compared with baseline. A subset of patients developed overt cardiotoxicity, and an additional proportion experienced borderline reductions in ejection fraction. Multivariable analysis identified prior trastuzumab-based chemotherapy and preexisting hypertension as independent predictors of LVD. In hypertensive patients previously treated with antihypertensive agents, this reflects the substantial cardioprotective benefits of these medications during anticancer therapy. These findings underscore the urgent need for intensive cardiac monitoring and proactive pharmacological management in breast cancer patients receiving targeted therapies, particularly those with underlying cardiovascular risk factors. To better understand the incidence, clinical results, and causal relationship between chemotherapy regimens and LVD, future research should incorporate sensitive biomarkers and imaging modalities, prospective long-term follow-up, and evaluate the cost-effectiveness of cardiotoxicity monitoring strategies alongside detailed assessments of concurrent cardioprotective interventions.

## Figures and Tables

**Table 1:** Characteristics of Study Subjects.

Data Type	Variable	Attribute/Unit	Value [n (%)]
Demographics	Age	Years (IQR)	47 (41.25–53)
		<40 years	53 (17.61)
		40–60 years	221 (73.42)
		>60 years	27 (8.97)
	BMI	kg/m^2^ (IQR)	24.92 (22.21–27.33)
		Underweight	11 (3.65)
		Normal	138 (45.85)
		Overweight	114 (37.87)
		Obesity	38 (12.62)
	BSA	m^2^ (IQR)	1.54 (1.47–1.64)
	Ethnicity	Javanese	87 (28.90)
		Sundanese	85 (28.24)
		Betawi	79 (26.25)
		Chinese	18 (5.98)
		Minangkabau	13 (4.32)
		Batak	10 (3.32)
		Malay	5 (1.66)
		Others	3 (1.33)
	Smoking history	Yes	1 (0.33)
Oncology	Cancer stage	Stage I	10 (3.32)
		Stage II	106 (35.22)
		Stage III	147 (48.84)
		Stage IV	38 (12.62)
	Left-sided tumor	Yes	153 (50.83)
	ER+ tumor	Yes	143 (47.51)
	PR+ tumor	Yes	134 (44.52)
	HER2+ tumor	Yes	123 (40.86)
	TNBC	Yes	69 (22.92)
	Received G-CSF	Yes	299 (99.34)
	Received radiotherapy	Yes	211 (70.10)
	Chemotherapy regimen	FAC	251 (83.39)
		AC	50 (16.61)
	Cumulative doxorubicin dose	mg/m^2^ (IQR)	360 (360–360)
		50–240 mg/m^2^	43 (14.29)
		50–360 mg/m^2^	258 (85.71)
	Subsequent chemotherapy	TCH	30 (9.97)
		TH	4 (1.33)
		None	267 (88.70)
	Trastuzumab cycles	Cycles (IQR)	0 (0–0)
		<18 cycles	17 (50.00)
		18 cycles	17 (50.00)
	Baseline LVEF	% (IQR)	65.60 (62.3–69)
Clinical	Hypertension	Yes	82 (27.24)
	Dyslipidemia	Yes	11 (3.65)
	Diabetes mellitus	Yes	7 (2.33)
	Atrial fibrillation	Yes	0 (0.00)
	History of PCI	Yes	2 (0.66)
	Renal failure (AKI/CKD)	Yes	0 (0.00)
Therapy	β-Blocker	Yes	21 (6.98)
	ACEI or ARB	Yes	45 (14.95)
	Statin	Yes	11 (3.65)
	Received cardioprotective therapy *	Yes	109 (36.21)

Note: (*) Cardioprotective therapy (ARB/ACEI, Diuretic, β-Blocker).

**Table 2:** Association of Variables with Left Ventricular Dysfunction.

Variable	Value			*p*-Value
	Non-Cardiotoxicity	Borderline Low	Cardiotoxicity	
	(n = 209; 69.43%)	(n = 72; 23.92%)	(n = 20; 6.65%)	
Age (IQR)	47 (42–54)	47.5 (41–51.5)	49 (40.75–51.25)	0.559 ^a^
<40 years	33 (15.79%)	14 (19.44%)	4 (20%)	0.890 ^b^
40–60 years	154 (73.68%)	52 (72.22%)	15 (75%)	
>60 years	22 (10.53%)	6 (8.33%)	1 (5%)	
BMI (IQR)	24.97 (22.35–27.27)	24.9 (22.54–28.13)	25.56 (21.12–27.39)	0.955 ^a^
Underweight	6 (2.87%)	3 (4.17%)	1 (5%)	0.859 ^b^
Normal	96 (45.93%)	33 (45.83%)	7 (35%)	
Overweight	80 (38.28%)	25 (34.72%)	9 (45%)	
Obese	27 (12.92%)	11 (15.28%)	3 (15%)	
BSA (IQR)	1.55 (1.48–1.64)	1.53 (1.45–1.62)	1.52 (1.46–1.61)	0.334 ^a^
Ethnicity				0.784 ^b^
Javanese	60 (28.71%)	19 (26.39%)	8 (40%)	
Sundanese	64 (30.62%)	15 (20.83%)	5 (25%)	
Betawi	55 (26.32%)	19 (26.39%)	5 (25%)	
Chinese	12 (5.74%)	6 (8.33%)	1 (5%)	
Minangkabau	7 (3.35%)	5 (6.94%)	1 (5%)	
Batak	6 (2.87%)	5 (6.94%)	0 (0%)	
Malay	3 (1.44%)	2 (2.78%)	0 (0%)	
Others	1 (0.48%)	1 (1.39%)	0 (0%)	
Smoking history	1 (0.33%)	0 (0%)	0 (0%)	0.802 ^b^
Cancer stage				0.648 ^a^
I	6 (2.87%)	3 (4.17%)	1 (5%)	
II	69 (33.01%)	27 (37.50%)	9 (45%)	
III	107 (51.20%)	31 (43.06%)	7 (35%)	
IV	27 (12.92%)	11 (15.28%)	3 (15%)	
Left-sided tumor	101 (51.67%)	40 (55.56%)	11 (55%)	0.518 ^b^
ER+ tumor	100 (47.85%)	30 (41.67%)	10 (50%)	0.644 ^b^
PR+ tumor	97 (46.41%)	28 (38.89%)	9 (45%)	0.514 ^b^
HER2+ tumor	85 (40.67%)	30 (41.67%)	9 (45%)	0.921 ^b^
TNBC	48 (22.97%)	16 (22.22%)	6 (30%)	0.758 ^b^
Received G-CSF	207 (99.04%)	72 (100%)	20 (100%)	0.642 ^b^
Received radiotherapy	144 (68.90%)	49 (68.06%)	16 (80%)	0.599 ^b^
Chemotherapy regimen				0.325 ^b^
FAC	172 (82.30%)	62 (86.11%)	19 (95%)	
AC	37 (17.70%)	10 (13.89%)	1 (5%)	
Cumulative doxorubicin dose (IQR)	360 (360–360)	360 (360–360)	360 (360–360)	0.450 ^a^
50–240 mg/m^2^	31 (14.83%)	9 (12.50%)	1 (5%)	0.557 ^b^
50–360 mg/m^2^	178 (85.17%)	63 (87.50%)	19 (95%)	
Subsequent chemotherapy				0.045 ^b^*
TCH	17 (8.13%)	6 (2.87%)	6 (30%)	
TH	4 (1.91%)	0 (0%)	0 (0%)	
None	188 (89.95%)	70 (97.13%)	14 (70%)	
Trastuzumab cycles				0.057 ^b^
<18 cycles	200 (95.69%)	3 (1.44%)	2 (10%)	
18 cycles	9 (4.31%)	3 (1.44%)	4 (20%)	
None	188 (89.95%)	70 (97.13%)	14 (70%)	
Baseline LVEF (IQR)	65% (62–67)	70% (67–74)	65.5% (61.28–68.23)	0.000 ^a^*
Medical History				
Hypertension	56 (26.79%)	24 (33.33%)	1 (5%)	0.029 ^b^*
Dyslipidaemia	10 (4.78%)	1 (1.39%)	0 (0%)	0.003 ^b^
Diabetes mellitus	4 (1.91%)	3 (4.17%)	0 (0%)	0.612 ^b^
History of PCI	2 (0.96%)	0 (0%)	0 (0%)	0.642 ^b^
Medication History				
β-Blocker	16 (7.66%)	5 (6.94%)	0 (0%)	0.684 ^b^
ACEI/ARB	32 (15.31%)	13 (18.06%)	0 (0%)	0.101 ^b^
Statin	10 (4.78%)	1 (1.39%)	0 (0%)	0.472 ^b^
Received cardioprotective therapy	58 (27.75%)	29 (40.28%)	5 (25%)	0.118 ^b^

Note: (*) Cardioprotective therapy (ARB/ACEI, Diuretic, β-Blocker); *p*-value < 0.05. (a) Kruskal–Wallis test. (b) Chi-Square test or Fisher’s exact.

**Table 3:** Multivariate Analysis of Hazard Ratios for Left Ventricular Dysfunction Events.

Variable	Univariate Analysis		*p*-Value	Multivariate Analysis		*p*-Value
	Hazard Ratio	95% CI		Adjusted Hazard Ratio	95% CI	
History of subsequent chemotherapy						
TCH and TH	3.94	1.45–9.59	0.009 *	3.79	1.39–6.45	0.011 *
Medical History						
Hypertension	0.20	0.02–0.78	0.017 *	0.20	0.81–5.39	0.02 *

Note: (*) *p*-value < 0.05.

**Table 4:** Hypertension Patients Receiving Cardioprotective.

Category Patient	n	Cardioprotective		*p*-Value
		No Medications	Received Medication	
Normal	220	170 (77.3%)	50 (22.7%)	2.32 × 10^-6 a^*
Hypertension	81	39 (48.1%)	42 (51.9%)	

Note: (*) Cardioprotective therapy (ARB/ACEI, Diuretic, β-Blocker); *p*-value < 0.05. (a) Chi-Square test.

## Data Availability

The data presented in this study are available on request from the corresponding author due to patient privacy concerns and ethical restrictions.

## Supplementary Materials

The following supporting information can be downloaded at: https://www.mdpi.com/article/10.3390/ph19060901/s1, Figure S1: Kaplan–Meier cardiotoxicity-free survival curves according to sequential trastuzumab exposure following doxorubicin-based chemotherapy; Figure S2: Kaplan–Meier cardiotoxicity-free survival curve for the overall study population during longitudinal follow-up.

## Abbreviations

The following abbreviations are used in this manuscript:

LVEF	Left Ventricular Ejection Fraction
GLOBOCAN	Global Cancer Observatory
HER2/ErbB2	Human Epidermal Growth Factor Receptor 2
LVD	Left Ventricular Dysfunction
ROS	Reactive Oxygen Species
ESC	European Society of Cardiology
IQR	Interquartile Range
BMI	Body Mass Index
BSA	Body Surface Area
ER	Estrogen Receptor
PR	Progesterone Receptor
TNBC	Triple-Negative Breast Cancer
FAC/AC	Fluorouracil, Adriamycin/Doxorubicin, Cyclophosphamide
TCH	Taxane, Cyclophosphamide, Trastuzumab
ACEI	Angiotensin-Converting Enzyme Inhibitors
ARB	Angiotensin Receptor Blockers
EF	Ejection Fraction
GLS	Global Longitudinal Strain
TIC	Trastuzumab-Induced Cardiotoxicity
HR	Hazard Ratio
_adj_HR	Adjusted Hazard Ratio
TOP2B	Topoisomerase II Beta
DNA	Deoxyribonucleic Acid
AIC	Anthracycline-Induced Cardiotoxicity
BSE	British Society of Echocardiography
CTRCD	Cancer Therapy-Related Cardiovascular Dysfunction
BNP	Brain Natriuretic Peptide
