# Supplementary material for: Trajectory and Determinants of Left Ventricular Dysfunction Following Anthracycline-Based Therapy Among Breast Cancer Patients: A Three-Year Cohort Analysis in Indonesia
Source: Pharmaceuticals (Basel). 2026 Jun 6;19(6):901. doi: 10.3390/ph19060901 (PMC13304912; doi:10.3390/ph19060901)
Supplement: Supplementary file 1 [file pharmaceuticals-19-00901-s001.zip › pharmaceuticals-4321700-supplementary.pdf]

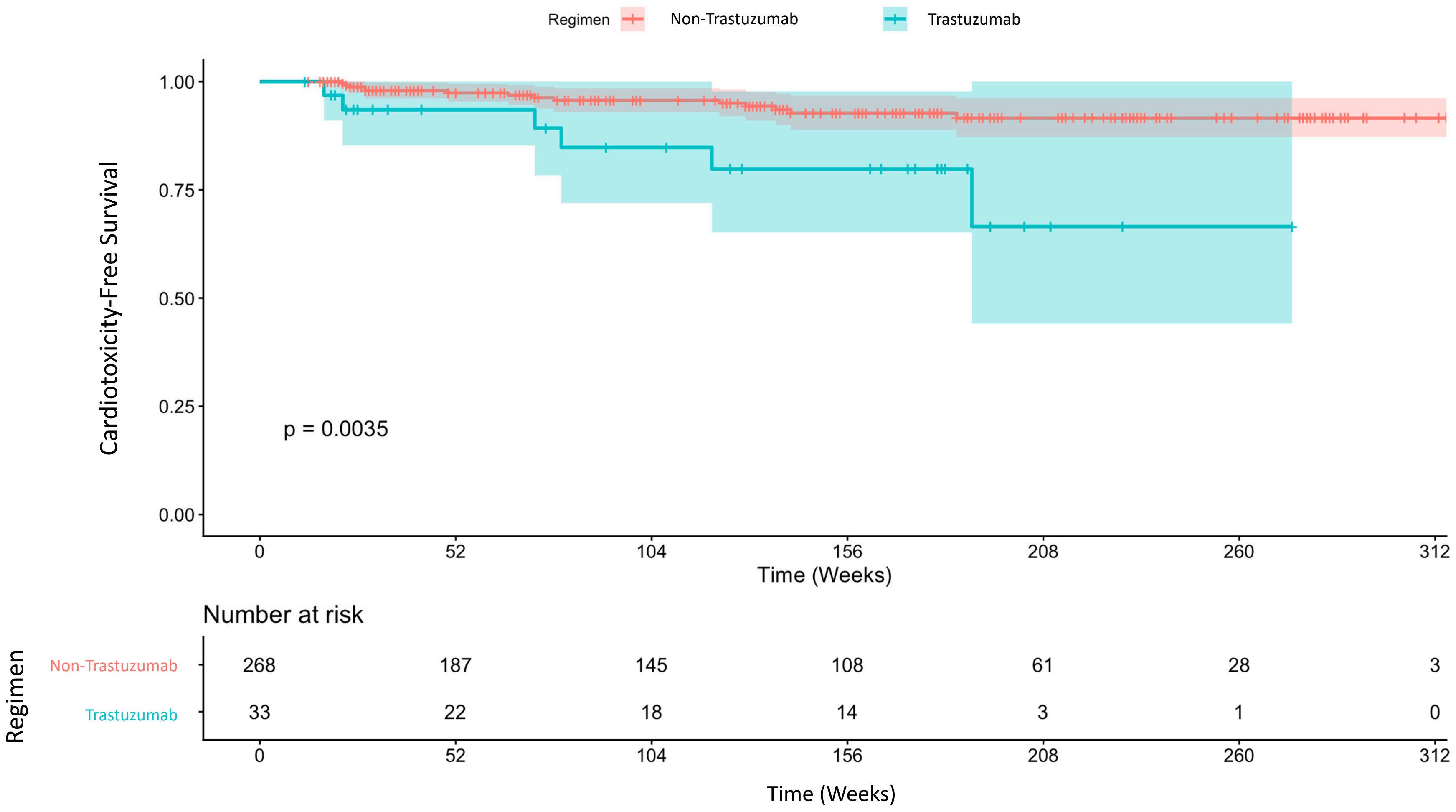

Supplementary Figure S1. Kaplan–Meier cardiotoxicity-free survival curves according to sequential trastuzumab exposure following doxorubicin-based chemotherapy.

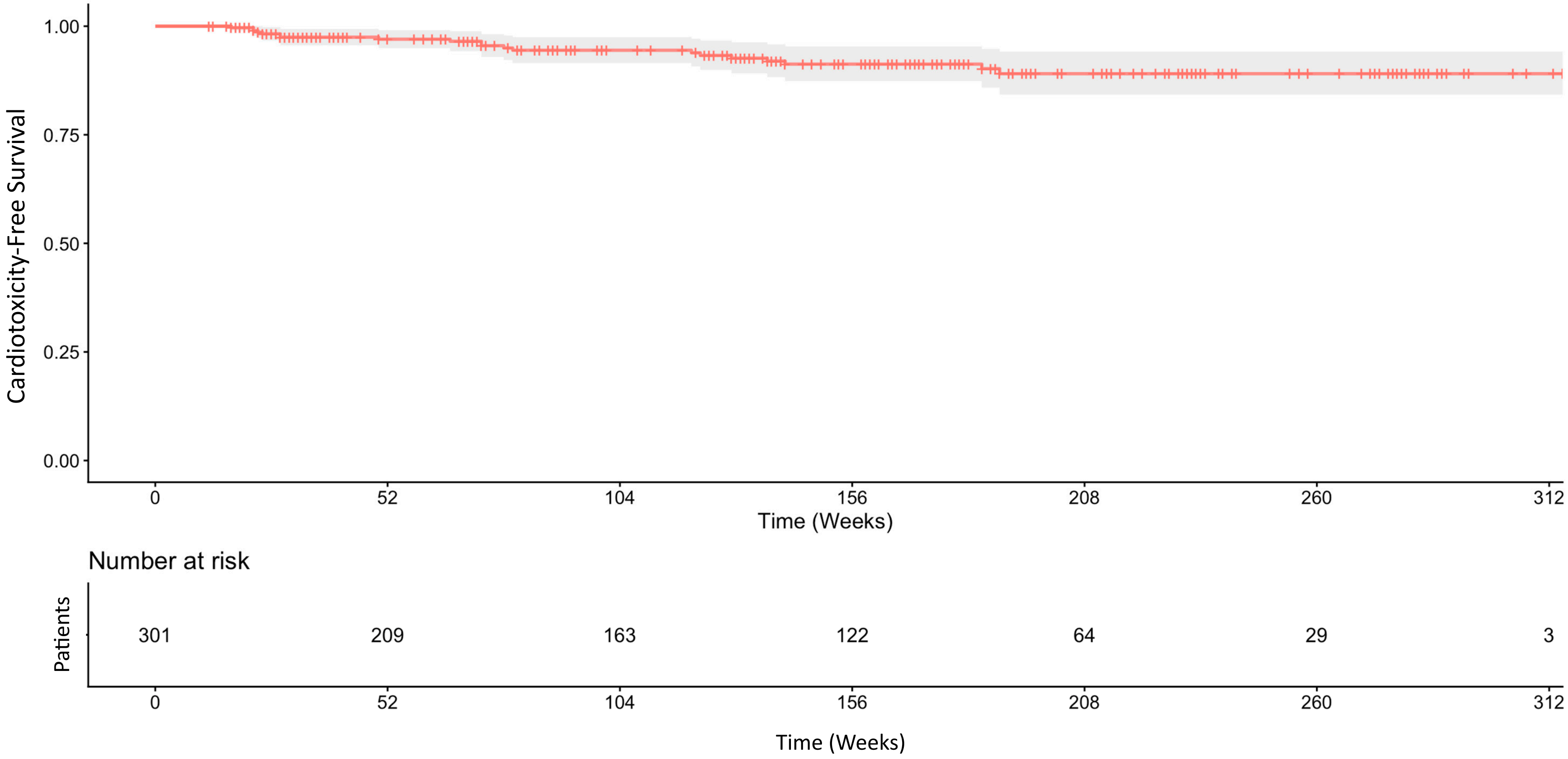

Supplementary Figure S2. Kaplan–Meier cardiotoxicity-free survival curve for the overall study population during longitudinal follow-up.
